# Insights into the Cross-Immunity Mechanism within Effector Families of Bacteria Type VI Secretion System from the Structure of *St*Tae4-*Ec*Tai4 Complex

**DOI:** 10.1371/journal.pone.0073782

**Published:** 2013-09-02

**Authors:** Heng Zhang, Zeng-Qiang Gao, Yong Wei, Jian-Hua Xu, Yu-Hui Dong

**Affiliations:** 1 Beijing Synchrotron Radiation Facility, Institute of High Energy Physics, Chinese Academy of Sciences, Beijing, People’s Republic of China; 2 School of Life Sciences, University of Science and Technology of China, Hefei, People’s Republic of China; Centre National de la Recherche Scientifique, Aix-Marseille Université, France

## Abstract

The Gram-negative bacteria type VI secretion system (T6SS) has been found to play an important role in interbacterial competition, biofilm formation and many other virulence-related processes. The bacteria harboring T6SS inject the effectors into their recipient’s cytoplasm or periplasm to kill them and meanwhile, to avoid inhibiting itself, the cognate immunity proteins were produced to acts as the effector inhibitor. Tae4 (type VI amidase effector 4) and Tai4 (type VI amidase immunity 4) are newly identified T6SS effector-immunity (EI) pairs. We have recently solved the structures of *St*Tae4-Tai4 and *Ec*Tae4-Tai4 complexes from the human pathogens *Salmonella typhimurium* and *Enterobacter cloacae*, respectively. It is very interesting and important to discover whether there is cross-neutralization between *St-* and *Ec*Tai4 and whether their effector inhibition mechanism is conserved. Here, we determined the crystal structure of *St*Tae4 in complex with *Ec*Tai4. The solution conformation study revealed it is a compact heterotetramer that consists of an *Ec*Tai4 homodimer binding two *St*Tae4 molecules in solution, different from that in crystal. A remarkable shift can be observed in both the flexible winding loop of *St*Tae4 and protruding loop of *Ec*Tai4 and disulfide bonds are formed to stabilize their overall conformations. The *in vitro* and *in vivo* interactions studies showed *Ec*Tai4 can efficiently rescue the cells from the toxicity of its cognate effectors *St*Tae4, but can not neutralize the toxic activities of the effectors from other families. These findings provide clear structural evidence to support the previous observation of cross-immunity within T6SS families and provide a basis for understanding their important roles in polymicrobial environments.

## Introduction

The type VI secretion system (T6SS) is a novel multi-subunit needle-like apparatus and plays an important role in many processes of bacterial life cycles, such as interspecies competition, biofilm formation and virulence-related processes [1]. The Gram-negative bacteria harboring T6SS inject the effectors into their recipient’s cytoplasm or periplasm to kill them. Meanwhile, to protect itself from accidental injury, the cognate immunity proteins were produced to protect the donor cells against the toxic effectors [2], [3]. Therefore, they can inhibit the growth of competitor cells without causing accidental injury to themselves and provide fitness advantages in the niche competition. Four broadly distributed and phylogenetically distinct families of T6SS peptidoglycan (PG) amidase effectors-immunity (EI) pairs have been recently identified based on overall primary sequence homology and different substrate specificities [4]. Tae4 (type VI amidase effector 4) and Tai4 (type VI amidase immunity 4) are T6SS effector-immunity pairs from the fourth family.

Our group has recently solved the crystal structures of *St-* and *Ec*Tae4-Tai4 complexes from the human pathogens *Salmonella typhimurium* and *Enterobacter cloacae*, respectively [5]. Structure-based mutational analysis of the *Ec*Tae4-Tai4 interface shows that a helix of one subunit in dimeric Tai4 plays a major role in binding Tae4, while a protruding loop in the other subunit is mainly responsible for inhibiting Tae4 activity. The inhibition process requires collaboration between the Tai4 dimer, distinctly different from that of Tse1 inhibiting by Tsi1 from the pathogen *Pseudomonas aeruginosa* (Tse1 and Tsi1 were recently renamed as Tae1 and Tai1, respectively) [4], [6], [7], [8]. Since *St-* and *Ec*Tae4-Tai4 complexes have similar structures, it is very interesting to discover whether there is cross-neutralization between the two immunity proteins within this family. Moreover, it is very important to find whether the mechanism of the effector inhibition and the key residues are conserved in the cross-neutralization process. To this end, we determined the high-resolution crystal structure of the effector *St*Tae4 from *S. typhimurium* in complex with the immunity protein *Ec*Tai4 from *E. cloacae* and studied the cross-neutralization of *Ec*Tai4 toward various effectors from the four families by *in vitro* and *in vivo* interactions. Our study has provided clear structural evidence to support the previous observation that there is cross-immunity within effector families of bacteria T6SS and provided a structural basis for understanding the essential roles of cross-immunity in polymicrobial environments.

## Results

### Overall Structure of *St*Tae4*-Ec*Tai4 Complex

**Figure 1:**
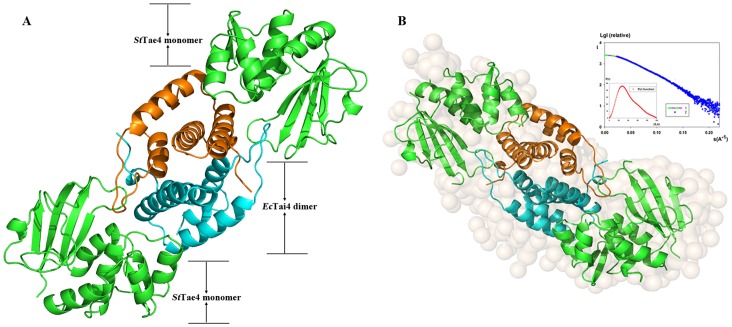
Structures of *St*Tae4-*Ec*Tai4 complex in crystal and solution. (A) Overall structure of *St*Tae4-*Ec*Tai4 complex (Also seen in Figure S1). The heterotetramer is composed of an *Ec*Tai4 homodimer [named subunit I (cyan) and subunit II (orange), binding two *St*Tae4 molecules in green. (B) Solution conformation of *St*Tae4-*Ec*Tai4 by SAXS analysis. Curve 1: experimental data. Curve 2: scattering patterns computed from the GASBOR model. Insertions: left below- P(r) function, right above-GASBOR models overlap with heterotetramer crystal structures. The experimental data compare well with the theoretical curves of crystal structure of *St*Tae4-*Ec*Tai4 complex.

Our initial attempts at solving the crystal structure of *St*Tae4*-Ec*Tai4 complex using the molecular replacement method with the known *St*Tae4 or *Ec*Tai4 structures as the searching model have not been successful. Then we solved the structure by the single-wavelength anomalous dispersion (SAD) method using Se-Met-labeled protein and refined it to a final *R*/*R*_free_ factor of 0.21/0.26 at 2.50 Å. The complex belonged to the *P2_1_2_1_2_1_* space group while the *Ec-* and *St*Tae4-Tai4 belonged to the *C121* and *P6_1_22* space group, respectively. There are sixteen molecules in the asymmetric unit of *St*Tae4-*Ec*Tai4 complex (Figures 1A and S1), while there are four and two molecules in that of *Ec-* and *St*Tae4-Tai4 (PDB code 4HFF and 4HFK), respectively. However, the retention volume of purified *St*Tae4*-Ec*Tai4 complex eluted from analytical size exclusion chromatography (Superdex 200) corresponded to a molecular mass of ∼57 kDa (Figure S2), which is much smaller than that of the total sixteen molecules above. To solve this contradiction, the small-angle X-ray scattering (SAXS) study, was applied to study the solution structure of the complex. As shown in Figure 1B, the fit of the theoretical curve of one tetramer crystal structure to the experimental data is very good fit with a discrepancy value of 1.625. The results indicate that the active complex is a heterotetramer in solution, consisting of a Tai4 homodimer [named subunit I (cyan) and subunit II (orange)] binding two Tae4 molecules (Figure 1A), consistent with the status of the *Ec-* and *St*Tae4-Tai4 complexes in solution. As previously demonstrated, molecular dimerization of *Ec*Tai4 is required in recognizing and binding *Ec*Tae4 [5], and the dimerization can also be observed in the present complex. The four heterotetramers in the asymmetric unit are likely the result of crystal packing (Figure S1), and represent no significant biological relevance. Since the four heterotetramers are essentially identical, we hereafter confine our analyses and discussions to one *St*Tae4*-Ec*Tai4 heterotetramer (composed of chains A and C for *St*Tae4 and chains B and D for *Ec*Tai4).

### Structural Comparisons of *St*Tae4/*Ec*Tai4 with their Respective Status in *St-* or *Ec*Tae4-Tai4 Complexes

**Figure 2:**
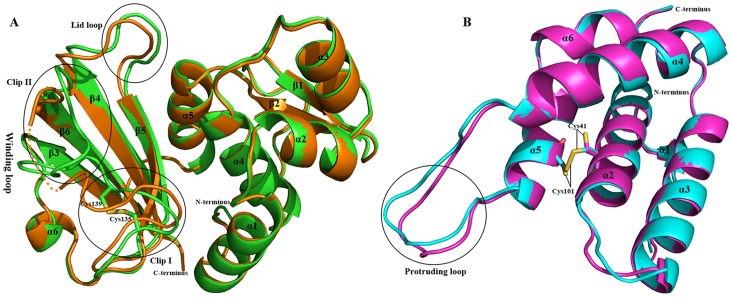
Structural comparisons of *St*Tae4/*Ec*Tai4 with their respective status in *St-* or *Ec*Tae4-Tai4 complexes. (A) Superposition of *St*Tae4 (green) in the present complex with that (orange) in the former *St*Tae4-Tai4. The residues from Leu142 to Gly148 in *St*Tae4 of *St*Tae4-Tai4 are without interpretable electron density in the crystal and are connected by dashed lines. The disulfide bond formed between Cys135 and Cys139 in the present *St*Tae4 of the present complex is shown in green sticks. The winding loop (composed of Clip I and II) and the lid loop are involved in the catalytic region. A remarkable conformation changes occurs in the winding loop interacting with *Ec*Tai4. (B) Superposition of *Ec*Tai4 (cyan) in the present complex with that (magenta) in *Ec*Tae4-Tai4. The residues Cys41 and Cys101 are shown in sticks, which form a disulfide bond in the present complex. A shift occurs in the protruding loop responsible for inhibiting the catalytic activity of Tae4.

The overall structure of *St*Tae4, especially the N-terminal (Nt) subdomain in the complex, is basically identical to that in *St*Tae4-Tai4 (PDB code 4HFF) and the catalytic triad Cys44-His126-Asp137 adopts very similar conformations in both structures (Figures 2A and S3). Structural alignments of *St*Tae4 with the former structure only give an RMSD of 0.415. However, there are clear differences in the C-terminal (Ct) subdomain. The most striking and divergent region is the disordered winding loop, which can be characterized as two clips (Figure 2A). Clip I, composed of the residues from Gly132 to Leu142, adopts similar structure to that in the former *St*Tae4, but a remarkable shift (∼5.4 Å) in the terminus occurs. It is worth to note that the conserved residues Cys135 and Cys139 form a disulfide bond (DSB) to stabilize the flexible loop, while there is no DSB between the two residues in the loop of the former one, where the two sulfydryl groups are in opposite directions (Figure 2A). As previously reported, the DSB is also observed in *Ec*Tae4 and Tae1, which provides their structural stability for substrate recognition and is closely associated with their PG amidase activities [4], [5]. The residues from Asn143 to Val151 form clip II, folding over the catalytic region. The clip II portion in *St*Tae4 is also remarkably different (a ∼4.8 Å shift) from that in the former one. The conformational flexibility of this clip has been proved to significantly affect the enzyme activity of *Ec*Tae4 [4]. The residues Leu142-Gln148 in Clip II of the winding loop observed in the present structure were missed in the former Tae4 of *St*Tae4-Tai4. Moreover, the overall conformation of the Clip II in the present structure is different from that in the former one from *St*Tae4-Tai4, which may be adaptive to the binding and inhibition of *Ec*Tai4. This indicates remarkable changes in the winding loop of *St*Tae4 will occur when recognized and inhibited by *Ec*Tai4 or other immunity proteins during the cross-immunity process. On the other hand, there are seldom changes in the closed lid loop covering the active pocket of Tae4 (Figure 2A), indicating the role of the loop in the inhibition process of *Ec*Tai4 against the active site of various effectors is similar.

The structure of *Ec*Tai4 in the present complex is also basically identical to that in *Ec*Tae4-Tai4 (PDB code 4HFK, Figure 2B). Structural alignments of *Ec*Tai4 with the former one give an RMSD of 0.468 Å. However, the dominant feature of the protruding loop in Tai4 is a shift by ∼3.0 Å compared with the former *Ec*Tae4 (Figure 2B), which may be adaptive for inserting into the active site of different effectors within this family. It is worth to note that the conserved residues Cys41 and Cys101, located in α2 and α5 respectively, form a DSB to stabilize the super helical conformation during the inhibition process, where there is no DSB formed between the two residues in the former *Ec*Tai4.

### Interaction of *St*Tae4 with Dimeric *Ec*Tai4

**Figure 3:**
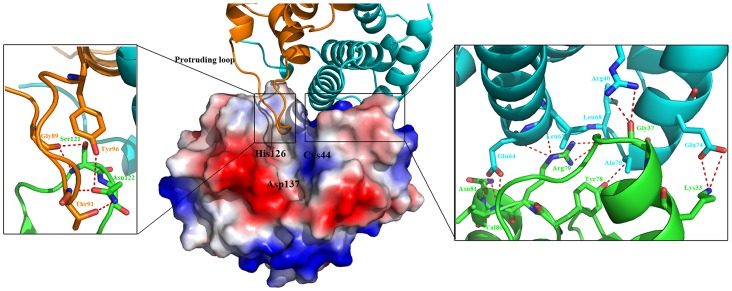
Binding and recognition of *St*Tae4 (shown as surface electrostatic potential, Middle) by *Ec*Tai4 dimer. Left and Right: the directly interacting residues between *St*Tae4 (in green cartoon) and *Ec*Tai4 subunits II (in orange) and I (in cyan), respectively. *Ec*Tai4 makes extensive contacts with *St*Tae4 and the protruding loop inserts into the active site containing the catalytic triad Cys44-His126-Asp137 of *St*Tae4.

There is intimate association between *St*Tae4 and *Ec*Tai4 homodimer in the interface (Figure 3 and Table S1). The total buried surface area in the interface of *Ec*Tai4 dimer with one *St*Tae4 monomer is 895 Å^2^, with 641 Å^2^ contributed by two helices (α3 and α5) from *St*Tae4 with two helices (α3 and α4) from *Ec*Tai4 subunit I, and 254 Å^2^ contributed by the lid loop from *St*Tae4 interacting with the protruding loop from the neighboring *Ec*Tai4 subunit II. A closer inspection of electrostatic potential mapped onto the molecular surfaces of *St*Tae4 and *Ec*Tai4 dimer, reveals a perfect surfaces complementary in both shape and electric charge (Figure 3, Middle), also suggesting there are extensive interactions between them. Notably, the highly conserved residues from Tyr78 to Asn81 in the loop β2-α5 and the helix α5 of *St*Tae4 directly interact with Ala70, Leu63, Glu64 and Leu68 of subunit I from *Ec*Tai4 through a series of hydrogen bonds; the residues Lys33 in the helix α3 of *St*Tae4 directly interacts with Glu74 of subunit I from *Ec*Tai4 through a salt bond (Figure 3, Right). On the other hand, the conserved residues Gly89, Thr91 and Tyr96 in the protruding loop of subunit II from *Ec*Tai4 forms direct interactions with the Ser121 and Asn122 in the lid loop of *St*Tae4 through hydrogen bonds (Figure 3, Left).

### Interaction Study between *Ec*Tai4 and different Effectors

**Figure 4:**
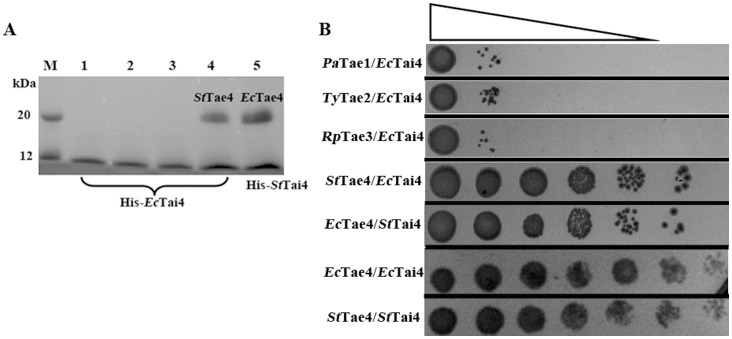
The *in vitro* and *in vivo* interactions studies. (A) Pull-down assays between His-*Ec*Tai4 and noncognate and cognate effectors. M, marker; 1, *Ec*Tai4-*Pa*Tae1; 2, *Ec*Tai4-*Ty*Tae2; 3, *Ec*Tai4-*Rp*Tae3; 4, *Ec*Tai4-*St*Tae4; 5, *St*Tai4-*Ec*Tae4. (B) Growth of *E. coli* co-expressing *Ec*Tai4 and various effectors above in the periplasm representing the cross-immunity between them. The cells were prepared with serial 10-fold dilutions from left to right.

The *in vitro* interactions of *Ec*Tai4 with the effectors *Pa*Tae1 (from *P. aeruginosa*), *Ty*Tae2 (from *Salmonella Typhi*), *Rp*Tae3 (from *Ralstonia pickettii*) or *St*Tae4 from different families of T6SS were studied by pull-down assays (Figure 4A). Our results showed that His-*Ec*Tai4 can pull down *St*Tae4,while His-*St*Tai4 can also pull down *Ec*Tae4 although we were unable to get the structure of the complex. However, His-*Ec*Tai4 can not pull down *Pa*Tae1, *Ty*Tae2 or *Rp*Tae3. The results indicate the immunity proteins may directly interact with their cognate effectors with the members of the fourth family, while there is no interaction between Tai4 and the effectors from other three families.

Co-expression of *Ec*Tai4 with the effectors above in periplasmic space of *E. coli* was further applied to test its neutralization capacity by observing the growth of *E. coli* (Figure 4B). The results showed *Ec-* and *St-*Tai4 can provide protection for the viability of *E. coli* harboring *St-* and *Ec-*Tae4, respectively, although their detoxification capacity is less lower compared with the native Tae4-Tai4 pairs. Meanwhile, *Ec-*Tai4 can not protect against the effectors from the other three families. This indicated both *Ec-* and *St-*Tai4 can efficiently rescue the cells from the toxicity of their cognate effectors within this family, but can not neutralize the toxic activity of the other family effectors.

## Discussion

### Conserved Inhibition Mechanism between *Ec*- and *St*Tai4

Although there are remarkable changes in the key regions of both *St*Tae4 and *Ec*Tai4, comparisons of the key residues in *Ec*Tai4 responsible for recognition and inhibition of *St*Tae4 with those in *Ec*Tai4-Tai4 showed they are conserved and very similar. These indicate the neutralization process of Tae4 among different species within the effector families of T6SS is similar, but the conformations of the flexible loops, which are associated with their enzyme activities, may vary as a result of the inhibition of different Tai4 from different species of this family. Moreover, more residues in *St*Tae4 are directly involved in binding and inhibiting *St*Tai4 compared with those in *Ec*Tai4 of the present complex. For example, the conserved residue Ser121 (or Arg124) in the lid loop of *St*Tae4 not only directly interacts Ala29 and Thr31 (or Tyr72) in the subunit I from *St*Tai4, but also with Asn96 in the protruding loop of subunit II in *St*Tae4-Tai4 complex. In the former *Ec*Tae4-Tai4 complex, the conserved residues E63A and E64A variants of *Ec*Tai4 cause a ∼140- and ∼10-fold reduction in affinity, respectively, indicating that these two residues are important for *Ec*Tae4 binding [5]. In the present complex, Leu63 directly interacts with Arg79, while Leu64 with Val80 and Asn81 of *St*Tae4, respectively (Figure 3, Right). We can reasonably speculate these two residues play similar role in the interaction with *St*Tae4. Besides, there is a direct interaction between Gly90 (in the protruding loop of *Ec*Tai4) and Ser151 (in the winding loop of *Ec*Tae4) in the former *Ec*Tae4-Tai4, while no direct interaction can be observed between these two loops in the present complex.

In the former *St*Tae4-Tai4 complex, there is a direct interaction by a hydrogen bond formed by Ser98 in the protruding loop of Tai4 and the catalytically important His126 in Tae4 via a water molecule (Figure S4). In the present structure and *Ec*Tae4-Tai4 complex, the tip of the protruding loop is also located near the catalytic triad. Moreover, although there is no direct interaction between the protruding loop of *Ec*Tai4 and the catalytic triad of *Ec-* or *St*Tae4, the variant Δ86-91 (the deletion from Gln86 to Thr91) in the protruding loop of *Ec*Tai4 has proved to be not capable of inhibiting amidase activity, but still can bind to *Ec*Tae4 [5]. In this complex, the residues Gly89, Thr91 and Tyr96 in the protruding loop of *Ec*Tai4 directly interact with Ser121 and Asn122 of *St*Tae4 through a series of hydrogen bonds (Figure 3, Left, and Table S1), in order to tether *Ec*Tai4 to the active site of *St*Tae4, in a similar mode to that in *Ec*Tai4-Tae4 complex. These results suggest the role of the protruding loop in inhibiting Tae4 is very similar among the family members.

### Structural Implications for the Cross-immunity of T6SS Effector Families

Not surprisingly, both *Ec-* and *St-*Tai4 can provide efficient protection for their cognate effectors *St-* and *Ec-*Tae4, but can not for the effectors from the other families. Similar observation has been reported that *Pa*Tai1 can rescue cells from the toxicity of both *Pa*Tae1 and the cognate *Bp*Tae1 (from *Burkholderia phytofirmans*), but can not neutralize the toxic activity of the effectors from other families [4]. These results showed all the species may avoid being killed from the toxicity of different effectors in the same family as a result of the intra cross-immunity. These interesting findings suggest a new strategy to develop anti-pathogen drugs. We can rationally design some small molecule inhibitors or small peptides that bind to the immunities, which could serve as a treatment against multiple pathogens within one effector families. However, an exception is that there no cross-immunity between *Ty*Tai2 and *Bt*Tai2 (from *Burkholderia thailandensis*) [4].

**Figure 5:**
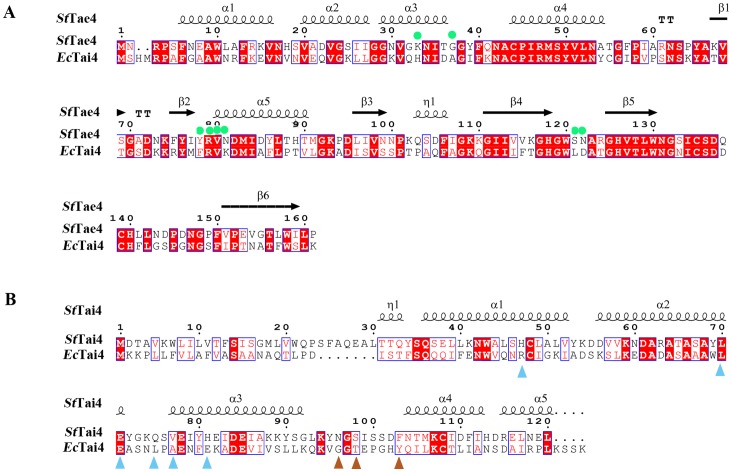
Structure-based sequence alignment for *St*Tae4 with *Ec*Tae4 (A), and *St*Tai4 with *Ec*Tai4 (B), performed using clustal X (version 1.81) and ESPript 2.2. The conserved residues are boxed in blue, identical conserved and low conserved residues are highlighted in red background and red letters, respectively. The directing residues in *St*Tae4 and *Ec*Tai4 of the present complex were shown in sphere (green) and triangle (cyan for subunit I and orange for subunit II, respectively).

In this study, both the amino acid sequences (with 53.4% identity, Figure 5A) and the structures (with a RMSD value of 0.574 Å) of *Ec*- and *St*Tae4 are very similar. More importantly, the structures of *Ec*- and *St*Tai4 are also similar with a RMSD value of 1.475 Å, although they share only 18.1% amino acid identity (Figure 5B). This structure-determined inhibition mechanism within this effectors family may be very conserved. Meanwhile, there is very low amino acid identity between Tae4 (or Tai4) and the other three families (Figure S5), and their structural arrangements are distinct. Tai1 and Tai4 are all-strand and all-helical protein [5], [6], [7], respectively, and Tai2 and Tai3 display α+β folds with different structural arrangements based on secondary structure prediction [9].The varied structures of the immunity proteins from different effector families are most likely to cause distinct inhibition mechanism of the effectors. Therefore, Tai4 can not provide efficient protection for the effectors from the other families. The structure of *Rp*Tai3 revealed its dimerization manner is different from its homolog *Bt*UCP from *Bacteroides thetaiotaomicron*, and the residues in the dimer interface are not conserved [9]. This indicated there may be different inhibition mechanisms within this effector family. Moreover, *Ty*Tai3 can neutralize the toxic activities of both *Ty*Tae3 and *Bt*Tae2, while *Bt*Tai2 can only provide protection for *Bt*Tae2 [4]. This indicates the cross-immunity is selective and the inhibition mechanisms of some members in these two families may be similar, as both of them have α+β structures. The variation of neutralization patterns shows the functional diversity of EI pairs.

### Cross-immunity of T6SS Effector Families in the Environment

Most of T6SS EI pairs are discovered in pathogens that colonize polymicrobial sites in the host and natural environment, such as the gastrointestinal tract (GI tract), and the soil [10], [11]. This suggests they are closely associated with interbacterial interactions in the formation of environmentally and clinically relevant microbial communities. Under these conditions, the cross-immunity against multiple effectors may promote the cooperation of some species and play an important role in interbacterial competition [10]. Moreover, Russell et al found 27% of the immunity proteins they identified were not encoded adjacent to intact effector genes, while the effector genes always co-occur with immunity genes [4]. This indicates there is a selective pressure to retain immunity even in the absence of cognate effectors for the antagonistic interspecies competition.

In this study, both *S. Typhimurium* and *E. cloacae* harboring the Tae4-Tai4 pair are common pathogens causing gastroenteropathy and they inhabit the polymicrobial environments in GI tract during most of their life cycle [12], [13]. Therefore, for competitions for survival in these environments by T6SS, their immunity proteins may be required to be retained as a result of a selective pressure. In the view of structural aspect combined with the interaction studies, our study proved *Ec-* and *St*Tai4 can provide the interactive immunity for both *Ec*- and *St*Tae4 and they can cooperate in the interbacterial competition, supporting the idea the necessity of retaining the immunity.

## Conclusion

The molecular insights into the *St*Tae4-*Ec*Tai4 complex structure and function garnered from this study shed light on the mechanisms of cross-immunity within effector families of T6SS. This work provides novel structural insights into the EI interaction, which is critical for accurately understanding the interplay between effector and immunity sequence variation, and assisting in our comprehension of bacterial interaction networks and community structure. Moreover, these findings provide valuable information for development of novel antibacterial agent that can control multiple pathogens.

## Materials and Methods

### Cloning, Expression, Purification and Crystallization

The genes encoding full-length *St*Tae4 and truncated *Ec*Tai4 (residues 19–117 without the N-terminal 18-residue signal peptide) were amplified from the *S. Typhimurium* and *E. cloacae* genomic DNA, respectively. The digested PCR products of *St*Tae4 was cloned into the *NcoI* and *XhoI* sites of pET28at-plus (introducing an N-terminal TEV cleavage site, constructed by our lab), while the digested PCR products of *Ec*Tai4 was cloned into the *NdeI* and *XhoI* sites of pET21a (Novagen, USA) with a C-terminal His tag. The two recombinant plasmids were co-transformed into BL21 (DE3) cells for co-expression. Recombinant proteins were purified as previously described [14].

The *St*Tae4-*Ec*Tai4 complex was concentrated to ∼15 mg/ml using Millipore Amicon Ultra 10 KD. Crystallization screens were performed with Hampton Research and QIAGEN kits using sitting-drop vapour-diffusion method at 293K. The SeMet complex crystal was obtained in the mixture solution containing 20% (w/v) PEG 3350 and 0.2 M Magnesium formate after 3 weeks.

### Data Collection, Structure Determination and Refinement

The diffraction data from a single crystal were collected on the beamline station BL17U1 of SSRF (Shanghai Synchrotron Radiation Facility) using an ADSC Q315r detector at a wavelength of 0.9792 Å. The total oscillation was 360° with 1° per image and the exposure time was 1 s per image. Before data collection, crystals were soaked for 5 s in a cryoprotectant consisting of 20% (v/v) glycerol in the crystal mother liquid and then flash-cooled in liquid nitrogen. The temperature was held at 100 K in cold nitrogen gas stream during data collection. The data were processed by HKL2000 [15]. At the first time, the space group was set to *P422* with unit cell parameters a = b = 89.36Å, c = 272.72 Å, however, the phases were too poor to get an interpretable electron density, so the space group was changed to the present one. The Se atoms were located by the program Shelxd [16], and then used to calculate the initial phases in Shelxe. The phases from Shelxe were improved in Resolve [17], and then used in Buccaneer for model building [18]. Coot and Phenix.refine were used for manually building and refinement, respectively [19], [20]. All the structures were validated by Molprobity [21]. Refinement statistics and model parameters were given in Table 1. The program PyMOL (http://www.pymol.sourceforge.net/) was used to prepare structural figures.

**Table 1:** Data collection and structure refinement statistics.

Data collection	
Wavelength (Å)	0.9792
Space group	P2_1_2_1_2_1_
Unit-cell parameters (Å)	a = 88.95, b = 89.13, c = 271.95
Resolution (Å)	2.50 (2.54–2.50)^a^
Number of unique reflections	75000(3723)
Completeness (%)	99.9 (100)
Redundancy	7.1(7.3)
Mean I/o' (I)	41.0(5.26)
Molecules in asymmetric unit	16
Matthews coefficient (Å^3^ Da^−1^)/solvent content (%)	2.35/47.7
R_merge_ (%)	9.20 (56.1)
**Refinement**	
Resolution range (Å)	46.2-2.50
R_work_/R_free_ (%)	21.0/26.1
No. of residues/protein atoms	2056/15755
No. of water atoms	220
Average B factor	
Main chain	49.66
Side chain	51.56
Waters	44.62
Ramachandran plot (%)	
Most favoured	97.3
Allowed	2.7
R.m.s. deviations	
Bond lengths (Å)	0.010
Bond angles (°)	1.246

a the values in parenthesis mean those of the highest resolution shell.

### Small-angle X-ray Scattering and Low Resolution Model Building

SAXS data were collected on the beamline station 1W2A in BSRF using a MARCCD165 detector. The scattering was recorded in the range of the momentum transfer 0.023<*s*<0.22 Å^−1^, in which *s = (4πsinθ)/λ*, *2θ* represents the scattering angle, and the X-ray wavelength *λ* is 1.54 Å. The measurements were performed in a cuvette (100 µl) with exposure time of 100 seconds to diminish the parasitic scattering.

The PRIMUS program was used to process the scattering curves [18]. The sample was measured at the concentrations of 1, 3 and 5 mg/ml to exclude concentration dependence. The distance distribution functions *p (r)* was computed with experimental data by the program GNOM [23]. The theoretical curves were calculated by the program CRYSOL [24]. The program GASBOR was used to build the *ab initio* low-resolution shapes of the complex in solution [25]. The protein structure is represented by an ensemble of dummy residues.

### Protein Pull-down Assay

The genes encoding *Pa*Tae1, *Ty*Tae2, *Rp*Tae3 and *St*Tae4 were amplified from the genomic DNA of *P. aeruginosa*, *S. Typhi*, *R. pickettii* and *S. Typhimurium*, respectively. The digested PCR products were cloned into the *NcoI* and *XhoI* sites of pET28at-plus. These recombinant plasmids were transformed in to *E. coli* strain for expression, respectively. His-tag *Ec*Tai4 and different effector proteins were treated with Ni beads at 277 K for 15 min. Subsequently, the native effector proteins were loaded into the beads, respectively. After extensive washing with 20 mM imidazole, the proteins were eluted with 250 mM imidazole and analyzed by SDS-PAGE and Coomassie blue staining.

### Cell Viability Assay

*Pa*Tae1, *Ty*Tae2, *Rp*Tae3 and *St*Tae4 were subcloned into the vector pET22b, while *Ec*Tai4 was subcloned into the vector pET26b. The *Ec*Tai4-pET26b was co-transformed into BL21 (DE3) cells with plasmids containing the effectors for co-expression in the periplasmic space. A single colony harboring the expressing plasmid was grown in LB media at 310K. After overnight culture, the cells were serially diluted in 10-fold steps and plated onto the LB agar supplemented with antibiotic and IPTG. The plates were prepared for pictures after an additional 20 h growth at 310 K. The strains harboring *St*Tae4-Tai4 or *Ec*Tae4-Tai4 were prepared as control.

### Protein Data Bank Accession Code

The atomic coordinates and structure factor files of *St*Tae4-*Ec*Tai4 complex have been deposited into the RCSB PDB with the code 4JUR.

## Supporting Information

Figure S1 **Four heterotetramers in the asymmetric unit of** ***St*** **Tae4-** ***Ec*** **Tai4 complex crystal.** The colors are shown as in Figure 1A.
(TIFF)

Figure S2 **Purified** ***St*** **Tae4-** ***Ec*** **Tai4 complex eluted from gel filtration chromatogram (superdex™ 200 10/300 GL) at 15.0 ml corresponded to a molecular mass of ∼57 kDa.**
(TIF)

Figure S3 **Superposition of the catalytic triad Cys44-His126-Asp137, Cys135 and Cys139 shown in sticks in** ***St*** **Tae4 (green) from the present complex with that (orange) from** ***St*** **Tae4-Tai4 complex.**
(TIF)

Figure S4 **Direct interaction between Ser98 in the protruding loop of Tai4 subunits II (in orange) and the catalytic His126 in Tae4 (in green) via a water molecule (W4, magenta) in the** ***St*** **Tae4-Tai4 complex.**
(TIF)

Figure S5 **Structure-based sequence alignment for** ***St*** **Tae4 with** ***Pa*** **Tae1,** ***Ty*** **Tae2 and** ***Rp*** **Tae3 (A), and** ***St*** **Tai4 with** ***Pa*** **Tai1,** ***Ty*** **Tai2 and** ***Rp*** **Tai3 (B), performed using clustal X (version 1.81) and ESPript 2.2.** The colors of the conserved residues were shown the same as Figure 5.
(TIF)

Table S1 **Detailed interactions between** ***St*** **Tae4 and** ***Ec*** **Tai4 homodimer.**
(DOC)
