# Supplementary figures and images for: Insights into the Cross-Immunity Mechanism within Effector Families of Bacteria Type VI Secretion System from the Structure of StTae4-EcTai4 Complex
Source: PLoS One. 2013 Sep 2;8(9):e73782. doi: 10.1371/journal.pone.0073782 (PMC3759425; doi:10.1371/journal.pone.0073782)

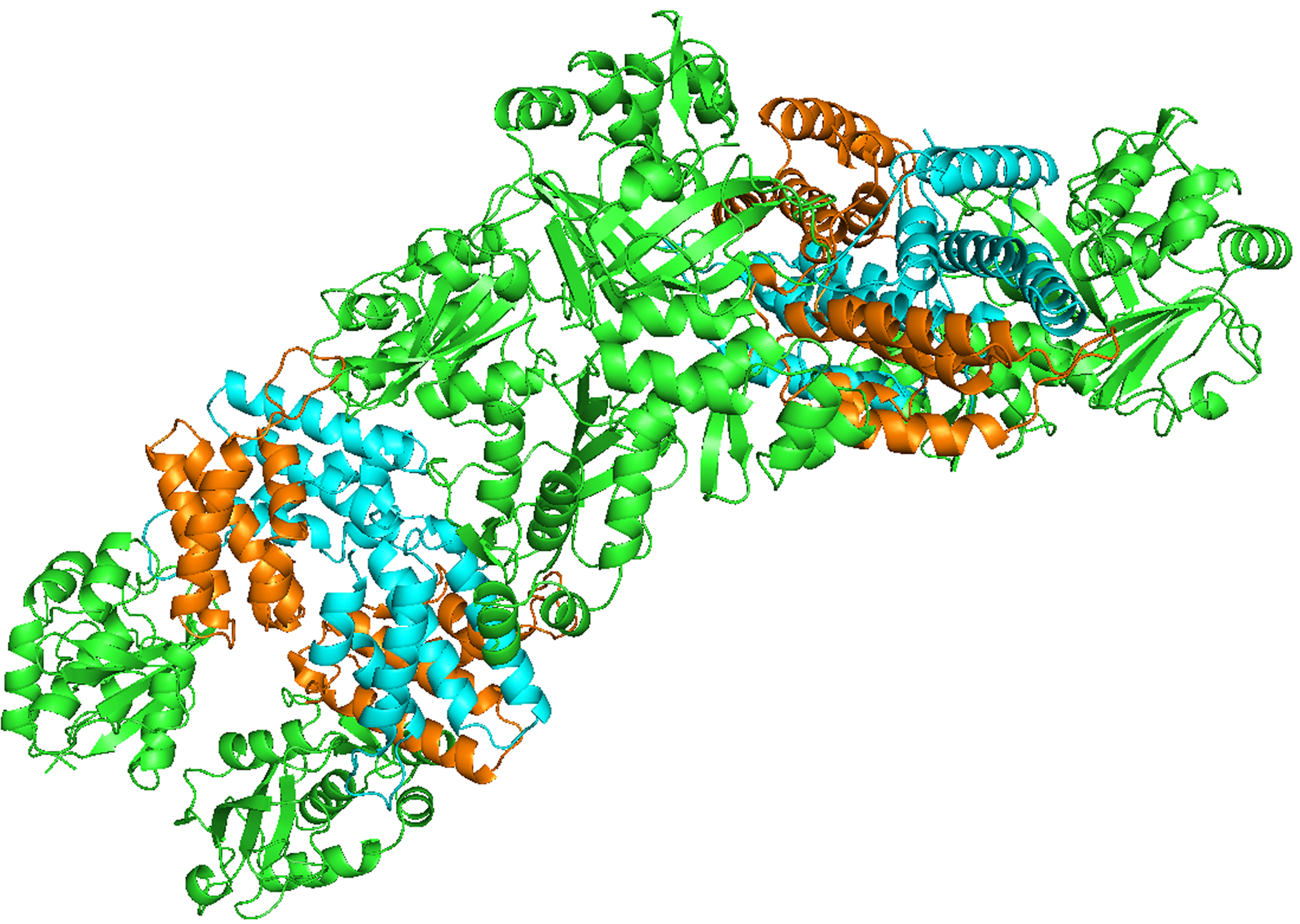

Supplement: Figure S1 — Four heterotetramers in the asymmetric unit of St Tae4- Ec Tai4 complex crystal. The colors are shown as in Figure 1A. (TIFF) [file pone.0073782.s001.tiff]

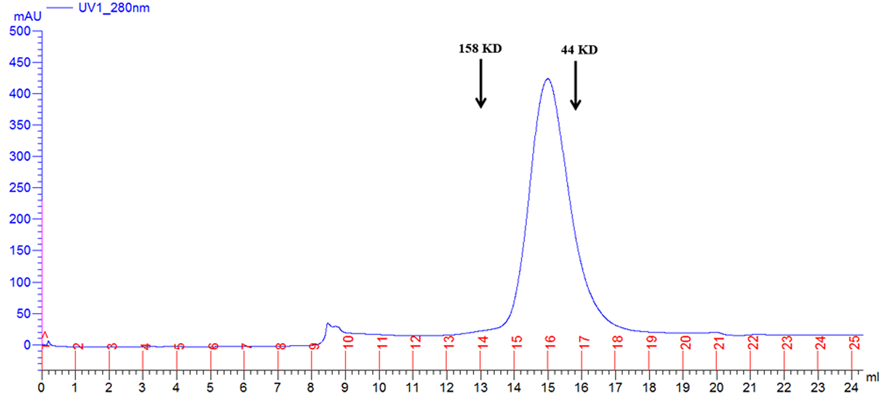

Supplement: Figure S2 — Purified St Tae4- Ec Tai4 complex eluted from gel filtration chromatogram (superdex™ 200 10/300 GL) at 15.0 ml corresponded to a molecular mass of ∼57 kDa. (TIF) [file pone.0073782.s002.tif]

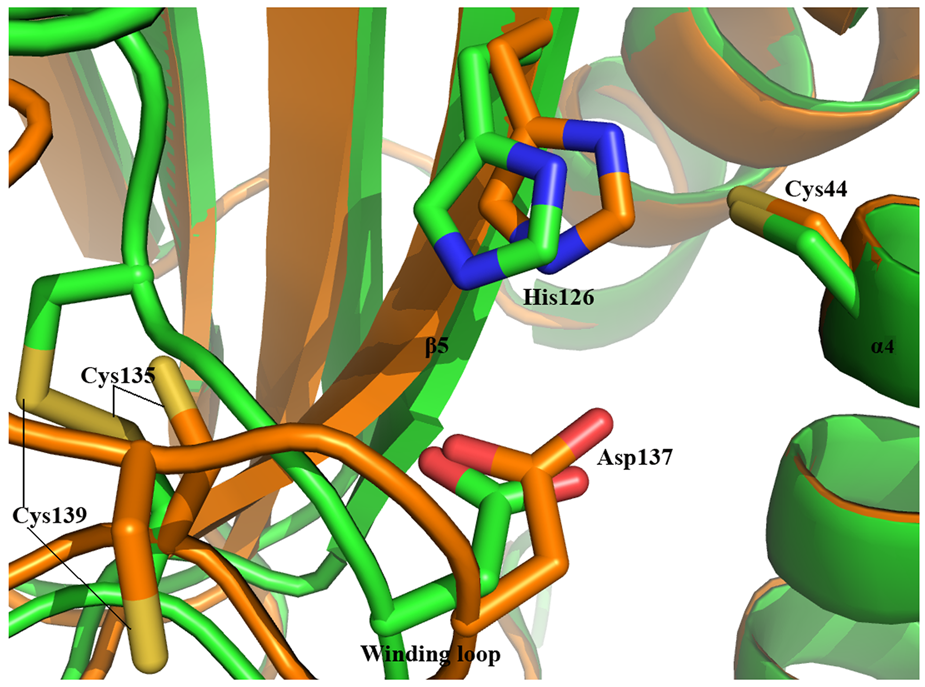

Supplement: Figure S3 — Superposition of the catalytic triad Cys44-His126-Asp137, Cys135 and Cys139 shown in sticks in St Tae4 (green) from the present complex with that (orange) from St Tae4-Tai4 complex. (TIF) [file pone.0073782.s003.tif]

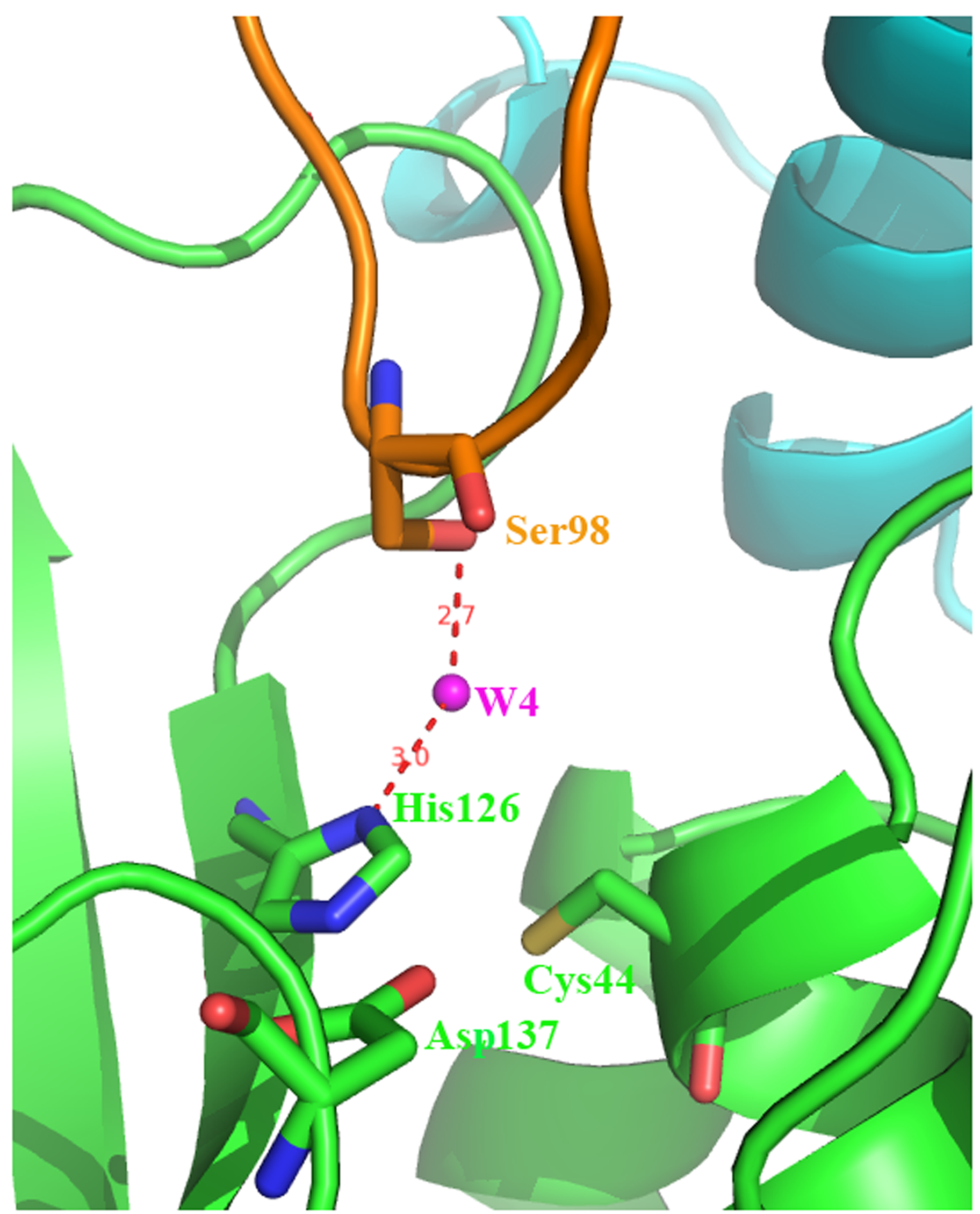

Supplement: Figure S4 — Direct interaction between Ser98 in the protruding loop of Tai4 subunits II (in orange) and the catalytic His126 in Tae4 (in green) via a water molecule (W4, magenta) in the St Tae4-Tai4 complex. (TIF) [file pone.0073782.s004.tif]

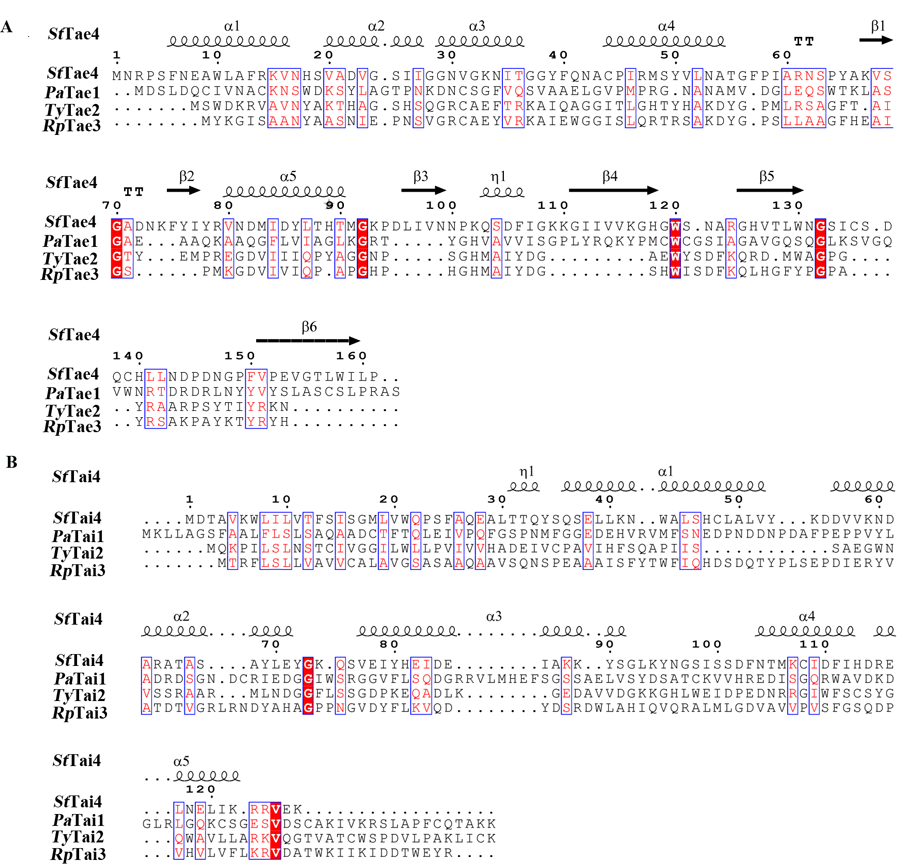

Supplement: Figure S5 — Structure-based sequence alignment for St Tae4 with Pa Tae1, Ty Tae2 and Rp Tae3 (A), and St Tai4 with Pa Tai1, Ty Tai2 and Rp Tai3 (B), performed using clustal X (version 1.81) and ESPript 2.2. The colors of the conserved residues were shown the same as Figure 5. (TIF) [file pone.0073782.s005.tif]
