# Supplementary material for: Insights into the Cross-Immunity Mechanism within Effector Families of Bacteria Type VI Secretion System from the Structure of StTae4-EcTai4 Complex
Source: PLoS One. 2013 Sep 2;8(9):e73782. doi: 10.1371/journal.pone.0073782 (PMC3759425; doi:10.1371/journal.pone.0073782)
Supplement: Table S1 — Detailed interactions between St Tae4 and Ec Tai4 homodimer. (DOC) [file pone.0073782.s006.doc]

**Table S1.** Detailed interactions between *St*Tae4 and *Ec*Tai4 homodimer.

| ***St*Tae4** | **Distance (Å)** | **Subunit I of *Ec*Tai4 dimer** |
| --- | --- | --- |
| Lys33 [NZ] | 3.11 | Glu74 [OE1] |
| Lys33 [NZ] | 3.56 | Glu74 [OE1] |
| Gly37 [O] | 3.28 | Arg40 [NH1] |
| Gly37 [O] | 3.01 | Arg40 [NH2] |
| Tyr78 [OH] | 3.04 | Ala70 [N] |
| Arg79 [NH2] | 2.88 | Leu68 [O] |
| Arg79 [NH2] | 3.12 | Leu63 [O] |
| Arg79 [NE] | 3.06 | Leu63 [O] |
| Val80 [N] | 3.22 | Glu74 [OE1] |
| Val80 [N] | 3.07 | Glu74 [OE2] |
|  | | |
| ***St*Tae4** | **Distance (Å)** | **Subunit II of *Ec*Tai4 dimer** |
| Ser121 [N] | 3.19 | Gly89 [O] |
| Ser121 [OG] | 2.68 | Gly89 [O] |
| Asn122 [ND2] | 2.64 | Thr91 [OG1] |
| Asn122 [OD1] | 2.96 | Thr91 [N] |
| Asn122 [OD2] | 3.82 | Tyr96 [OH] |
